# Nutrition and Iron Status of 1-Year Olds following a Revision in Infant Dietary Recommendations

**DOI:** 10.1155/2011/986303

**Published:** 2011-07-18

**Authors:** Asa V. Thorisdottir, Inga Thorsdottir, Gestur I. Palsson

**Affiliations:** ^1^Unit for Nutrition Research, Landspitali-The National University Hospital of Iceland, Eiríksgata 29, 101 Reykjavik, Iceland; ^2^Faculty of Food Science and Nutrition, University of Iceland, 101 Reykjavik, Iceland; ^3^Children's Hospital, Landspitali-The National University Hospital of Iceland, 101 Reykjavik, Iceland

## Abstract

A previous study showed low iron status in 12-month-old Icelandic infants associated most strongly with cow's milk intake and growth. Infant dietary recommendations were revised in 2003. This study investigated nutrition and iron status in a new infant cohort. *Subjects/Methods*. Randomly selected infants were prospectively investigated for diet, anthropometry, and iron status (*n* = 110–141). *Results*. Breastfeeding initiation rate was 98%; 38% of 5-month olds were exclusively and 20% of 12-month olds partially breastfed. Formula was given to 21% of 6-month olds and 64% of 12-month olds, but cow's milk to 2.5% and 54.4% of 6- and 12-month olds, respectively. Iron depletion (serum ferritin < 12 *μ*g/L) affected 5.8%, 1.4% were also iron deficient (MCV < 74 fl), and none were anemic (Hb < 105 g/l). Iron status associated negatively with growth and breastfeeding duration and positively with meat and formula intake at 9–12 months, but not with cow's milk. *Conclusion*. Improved iron status might be explained by a shift from cow's milk to formula in the diet of Icelandic 6–12-month olds. Dietary changes altered associations between foods and iron status.

## 1. Introduction 

Adequate iron status in infancy is important because of its effects on health and development [1]. Infants are especially susceptible to iron deficiency (ID) and iron deficiency anemia (IDA) because exogenous iron requirements increase rapidly during the second half of the first year due to high growth rate in infancy. 

Iron status in infancy has been negatively associated with consumption of cow's milk [2, 3] and positively with meat intake [2, 4–6]. A prospective study on Icelandic infants in 1995–1997 also found these associations where 20% of 12-month-old Icelanders had ID (mean corpuscular volume (MCV) < 74 fl, serum ferritin (SF) < 12 *μ*g/L), 2.7% had IDA (hemoglobin (Hb) < 105 g/L, MCV < 74 fl, SF < 12 *μ*g/L), and 41% were iron-depleted (SF < 12 *μ*g/L) [7]. The iron status of Icelandic infants was considerably worse compared to similar infant populations in the nineties in Denmark [2], Sweden [4], and Norway [8]. Iron-deficient 12-month olds had been breastfed 2.5 months shorter than nondeficient infants, and a multiple regression analyses revealed that the effect on iron status was almost universally accounted for by intake of regular cow's milk at 9 and 12 months of age [7]. Higher growth velocity during the first year had an independent negative association with iron status at 1 year (y). Furthermore, low iron status was observed to negatively affect later growth and iron status [9] as well as motor developmental scores in 6-year olds in a developed affluent society such as Iceland [10]. 

In 2003 revised infant dietary recommendations were published in Iceland, where iron-fortified formula was recommended in the weaning period from six months of age [11]. The formula, Icelandic follow-on milk, was made available in every grocery shop ready-made in cartons at a fair price. Breastfeeding was emphasized more than before by adopting the World Health Organization (WHO) recommendation for exclusive breastfeeding until six months of age [12] instead of 4–6 months as recommended previously and also by encouraging partial breastfeeding, preferably until 1-year old or longer if it suits mother and child. Compared to regular cow's milk, the iron-fortified formula has higher iron (0.75 mg versus 0.023 mg/100 g) and lower protein (1.8 g versus 3.4 g/100 g) concentration and is complemented with other nutrients such as vitamin C (9 mg/100 g) [13] to fulfill the Codex Alimentarius, Regulation no. 735/1997 [14]. The iron content is modest and in accordance with other milk-based formulas supporting normal growth and iron status of healthy infants [15]. The revised recommendations are provided to all parents of newborns by healthcare professionals at healthcare centers. Fortification is thought to be the most effective strategy to combat nutritional deficiencies, but nutrition education interventions are also effective in improving iron status in children [16] as well as screening for iron deficiencies in more vulnerable populations [17].

The objective of this study was to investigate a prospective cohort of 12-month olds, where data collection occurred after implementation of the revised infant dietary recommendations, to evaluate iron status and its association with diet and growth. Moreover, we aim to assess the impact of the revised recommendations on infants' food and nutrient intake and iron status.

## 2. Subjects and Methods

### 2.1. Sample Recruitment

A random sample of 250 Icelandic infants born in 2005, from January to December, was collected by Statistics Iceland. The criteria for participation were the same as in the former prospective study on infant nutrition 1995–1997 [7]. The criteria were Icelandic parents, singleton birth, gestational length of 37–41 weeks, birth weight within the 10th–90th percentiles, no birth defects or congenital long-term diseases, and the mother had early and regular antenatal care. Eligible participants were 196. Informed written consent from the parents was obtained, and all individual information was processed with strict confidentiality. The study was approved by the National Bioethical Committee in Iceland (VSNb2005040019/037) and registered at the Icelandic Data Protection Authority (S2449/2005).

### 2.2. Dietary Assessment

Dietary data from 0–4 months of age was collected by dietary-history, including questions on breastfeeding, infant formula-feeding, other food items, and supplements. Food records were filled out monthly by the parents. Weighed food records were kept for three consecutive days (72 hours) at 9 and 12 months. All food was weighed on accurate electronic scales (PHILIPS HR 2385, Hungary; ±1 g accuracy). The breastfed infants were weighed with the same clothes before and after each breastfeeding session (Tanita model 1583, Japan or Sega model 336 7021099, Germany; both with ±10 g accuracy) to estimate the amount of breast-milk consumed as formerly described [18]. A nutrient calculation program, ICEFOOD, was used to calculate the weighed food records [19]. At 5–8 and 10-11 months of age intake was estimated by 24-hour food recall using common household measures, such as cups and spoons.

### 2.3. Biochemical Analysis and Anthropometric Measures

At 12 months of age blood samples were obtained for analyzing iron status. Hb, MCV, and SF were analyzed on the Coulter Counter STKS at Landspitali-The National University Hospital of Iceland. The definition for IDA includes all three indicators below cutoff points, Hb < 105 g/L, MCV < 74 fl, and SF < 12 *μ*g/L, for ID the two latter indicators are below cutoff points and iron depletion as having SF < 12 *μ*g/L [2, 7, 20, 21]. Anthropometry, that is, weight, length, and head circumference at birth, 1, 2, 3, 5, 6, 8, 10, and 12 months, was recorded at maternity wards and healthcare centers.

### 2.4. Statistical Analysis

Statistical analyses were performed with SPSS software (version 17; SPSS INC, Chicago, IL). Descriptive statistics were used for the participants' characteristics and food and nutrient intake, presented as mean and standard deviation (SD) for normally distributed variables and median with interquartile range (IQR) for skewed variables. For comparison between two groups, independent *t*-test, Mann-Whitney *U* test, or chi-square was used.

To identify predictors of the infants' iron status indices (Hb, SF, and MCV) multivariate regression models were constructed. Variables which differed between iron-depleted infants and non-iron-depleted infants were used in the model for SF. Because of skewed distribution, SF was logarithmically transformed. The model for each iron status index also included their correlating variables (Pearson's correlation coefficient), and for interrelated variables those with the weakest correlation to each iron status index were excluded from the model. Birth weight was adjusted for in the models. The sample size of 200, expecting 30% dropout, gives a power of 90% to detect the effect of 100 g weight gain on SF. To detect difference in iron status between the former (1995–1997) and current (2005–2007) cohorts the power was >90%. The level of significance in the study was *P* ≤ .05.

## 3. Results

### 3.1. Study Group

A total of 141 infants (73 boys and 68 girls) participated in the study, 72% of the 196 eligible participants. The characteristics of the subjects and their parents are presented in Table 1. Dropouts did not differ significantly from participants in basic values, that is, size at birth, 3698.8 (340.6) g versus 3781.3 (368.2) g (*P* = .078), and exclusive breastfeeding duration, 3.2 (1.8) months versus 3.54 (1.8) months (*P* = .073). Characteristics, iron status and anthropometry, did not differ between children with complete (*n* = 110) and incomplete (*n* = 31) dietary data, that is, Hb values 120.4 (7.7) g/L versus 121.3 (9.8) g/L (*P* = .658), SF values 36.6 (22.1) *μ*g/L versus 35.1 (19.7) *μ*g/L (*P* = .802), or weight at 12 months 10038.5 (1018.4) g versus 10229.8 (1125.5) g (*P* = .413). 

### 3.2. Diet and Nutrient Intake

In the present study 97.8% of mothers initiated breastfeeding and 38.1% of 5-month olds and 7.2% of 6-month olds were exclusively breastfed and 20.0% were still partially breastfed at 12 months of age. Average food intake was similar among boys and girls, except for milk consumption (Table 2). A higher proportion of boys were exclusively breastfed at 6 months and partially at 12 months of age. A higher proportion of girls received formula at 9 and 12 months of age but consumption of regular cow's milk did not differ between the genders (Table 2). The mean (SD) intake of formula for 9–12-month olds was 156 (14.9) g/day and only 43 (9.2) g/day for regular cow's milk. Vitamin D supplementation was higher among boys than girls at 6 months of age, 60.2% among boys versus 48.1% among girls (*P* = .051). Fruit consumption was more common than vegetable consumption among 5–8-month olds, 85% or more of 6–8-month olds consumed porridge, and dairy product consumption (excluding drinking milk or formula) was modest; 1.2% of 5-month olds and 26.8% of 8-month olds consumed dairy products of some sort. The average energy intake among the subjects was close to the estimated average daily requirements (kJ/kg of body weight). The protein intake, however, exceeded the recommendations for this age group (Table 3). Nutrient intake did not differ significantly between boys and girls except for vitamin A intake at 12 months of age, which was higher for girls.

### 3.3. Iron Status and Associations with Diet, Growth, and Sociodemographic Factors

There were no children with IDA. ID affected 1.4% (*n* = 2) of the children, and 5.8% (*n* = 8) of the children were iron depleted. No significant gender difference was found on MCV and Hb, but girls had significantly higher SF level, 39.2 (17.5) *μ*g/L, compared to, 33.5 (24.6) *μ*g/L, boys (*P* = .003). 

Iron-depleted infants had lower birth weight, 3402 (255.8) g versus 3805 (361.7) g (*P* = .002), and higher growth rate than non-iron-depleted ones. Comparing iron-depleted and non-iron-depleted infants, proportional weight gain was 2.04 (0.25) versus 1.66 (0.34) (*P* = .003), absolute weight gain 4.8 (0.6) kg versus 4.3 (0.8) kg (*P* = .043) from 0 to 6 months, and 6.9 (0.8) kg versus 6.3 (1) kg (*P* = .061) from 0 to 12 months, and length growth from 0 to 12 months was 18.5 (0.82) cm versus 17.0 (1.9) cm (*P* = .028). The iron-depleted infants had longer breastfeeding duration, 9.4 (3.5) months versus 7.6 (3.3) months, although not significant, and consumed less formula, 10.5 (16.3) g/day versus 133.0 (159.9) g/day at 9 months (*P* = .038) and 33.5 (47.2) g/day versus 187.3 (199.9) g/day at 12 months (*P* = .044). Dietary vitamin and mineral intake did however, not differ significantly between iron-depleted and non-iron-depleted infants. 

SF level associated negatively with growth variables in infancy, but positively with birth weight. For growth from 0 to 12 months of age proportional weight gain (which takes birth weight into account) and absolute length gain correlated most negatively with SF level. For growth from 0 to 6 months weight gain correlated most negatively with SF, but growth from 6 to 12 months did not correlate significantly with the variable. After adjusting for birth weight multiple regression analysis showed that log SF level in boys decreased 0.0201 *μ*g/L for each 100 g of weight gain from 0 to 6 months, 0.0174 *μ*g/L for each 100 g of weight gain from 0 to 12 months and 0.0823 *μ*g/L for each cm of length gain from 0 to 12 months (Table 4). In girls, iron-fortified formula, porridge (positive), and bread (negative) consumption associated independently with log SF level after adjusting for birth weight (Table 4). 

Hb level had weaker associations with growth variables (positive) than SF level. In boys Hb associated most positively with dietary iron and meat consumption and most negatively with breastfeeding duration; formula had a weaker but significant association with Hb (positive). In girls fruits and dairy products were most strongly independently associated with Hb level (Table 4). Hb level was lower in infants exclusively breastfed for 5 months or longer than in those exclusively breastfed for less than 5 months (118.6 g/L versus 121.6 g/L, *P* = .034). Exclusive breastfeeding was excluded from the regression model because partial breastfeeding duration was a stronger predictor of Hb level, and the two variables were strongly intercorrelated. 

MCV did not associate with any growth variable. In boys the only variable associated independently with MCV, of borderline significance, was breastfeeding duration (negative). In girls MCV level associated most positively with dietary iron intake and a weaker association was found with dietary vitamin C intake. 

No associations were found between iron status indices and sociodemographic variables, that is, habitation, parents' education, age, BMI, or smoking habits.

## 4. Discussion

The present study describes nutrition and iron status in well-nourished infants with low prevalence of ID and IDA, high birth weight, and breastfeeding rate. Dietary intake in infancy has changed towards the infant dietary recommendations, revised in 2003, and iron status has improved when compared to results from an earlier prospective study of a comparable nationwide cohort. Breastfeeding has increased but the main alteration in the diet is an iron-fortified formula which has replaced regular cow's milk in the latter half of the first year. The altered diet seems to have deleted cow's milk as the main variable influencing serum ferritin and iron status. The formula was developed from domestic cow's milk to diminish the change for the infants' population as Icelandic cow's milk has some characteristics different from major brands [22]. Calculated nutrient intake showed that mean intake of iron, zinc, and calcium was below RDI. However, iron status was acceptable irrespective of the low iron intake. This may partially be explained by incomplete nutrient information in the database, such as for organic infant porridges which were commonly consumed by the study population. The RDI is estimated to meet the needs of 97.5% of the total population and RDI for zinc and calcium is extrapolated from the adult RDI, which can result in unnecessarily high recommendations for infants. Therefore the risk of deficiency might be small. However, further studies are needed on young children's vitamin and mineral status. 

**Figure 1:**
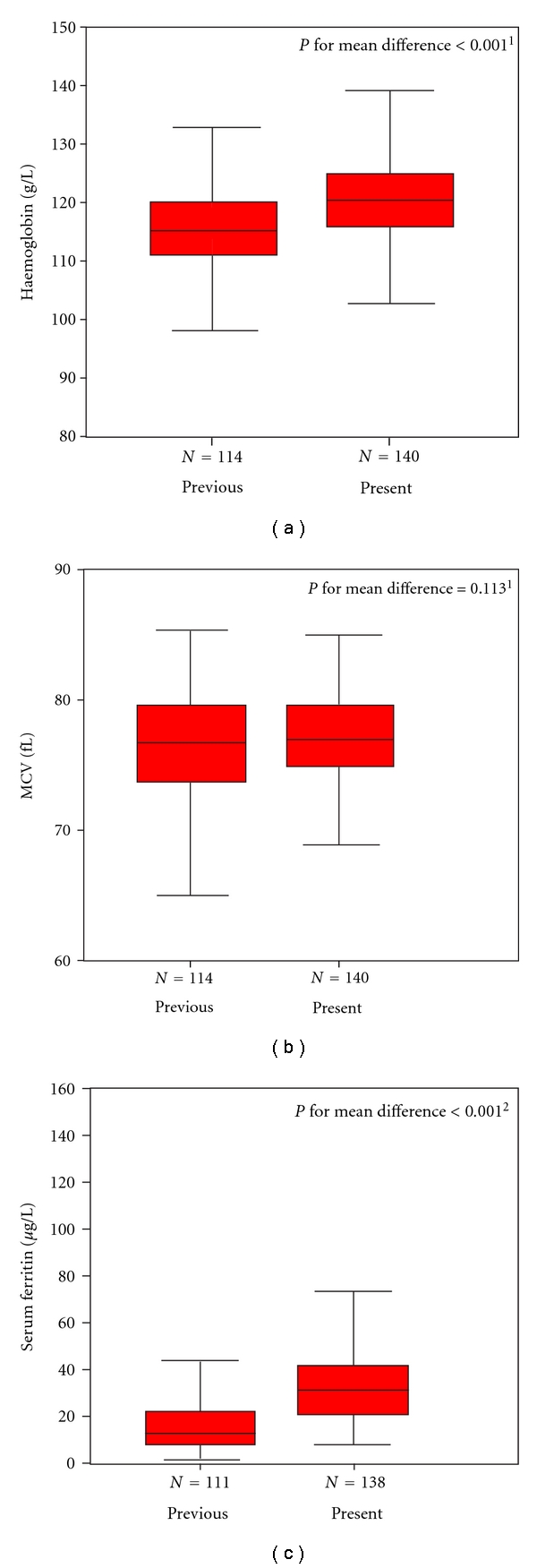
Box plots of iron status indices in 12-month olds, from previous (1995–1997) and present (2005–2007) studies. The box plots show the median, quartiles, maximum, and minimum values. Independent *t*-test was used for hemoglobin (*P* = .001), and MCV (*P* = .113), data were normally distributed and Mann-Whitney test for serum ferritin (*P* = .001) data were not normally distributed.

In comparison with the former study on infant nutrition (1995–1997), iron status of 12-month-old Icelandic infants has improved significantly (Figure 1). The prevalence of ID decreased from 20% to 1.4%, iron depletion from 41% to 5.8%, and IDA from 2.7% to 0. The participants in the two studies were nationwide random samples of healthy term infants and analogous with respect to sociodemographic factors, that is, habitation, number of siblings, parents' age, and education [7], and therefore comparable. Iron status in the 1995–1997 infant study was worse than in the neighboring countries [7] but according to the latest data from the Scandinavian countries there seems to be a lower prevalence of ID infants in Iceland than in the other Nordic countries. In a Swedish and a Norwegian study 18% and 10% of 12-month-old infants, respectively, were iron depleted (SF < 12 *μ*g/L) [4, 8], compared with 5.8% in the 2005–2007 infant cohort now presented. In the present study the threshold for Hb level was 105 g/L and for MCV it was 74 fl, as it was also used in the previous Icelandic infant study and other Nordic studies for this age group [2, 7, 20, 21]. The thresholds for infants are, however, controversial, and the World Health Organization recommends the use of 110 g/L for Hb in children between the ages of 6 to 59 months and 79 fl for MCV in children between the ages of 1 to 1.9 years. Changing the thresholds for Hb and MCV in our cohorts towards WHO standards did not change the result or the conclusion of the study, the difference between the two cohorts was even larger. 

**Figure 2:**
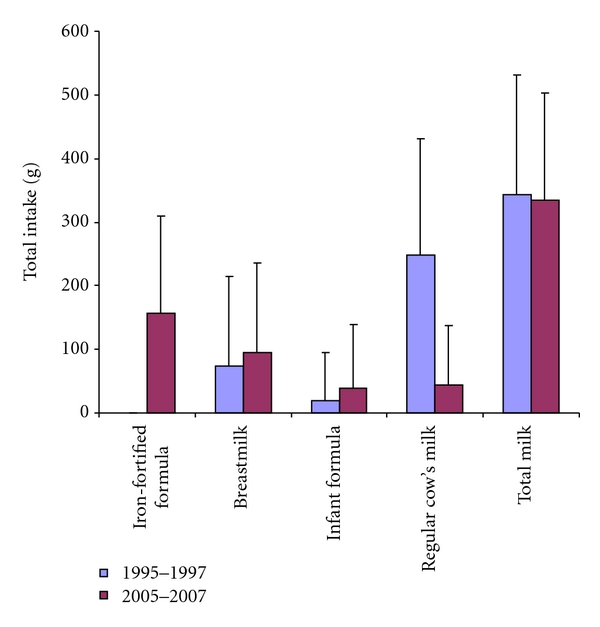
Difference in milk consumption between previous, (1995–1997) and present (2005–2007) studies, showed as mean and SD.

In the present study the negative association between iron status and regular cow's milk seen in the 1995–1997 infant study had disappeared as the consumption had decreased and been replaced by the iron-fortified formula (Figure 2). Iron-fortified formula had a weak positive association with iron status. Breastfeeding rates have increased since the previous study, both partial and exclusive breastfeeding, but exclusive breastfeeding rates among 6-month olds are still low. That may partly be explained by the fact that the data for exclusive breastfeeding was collected from the recorded food intake at monthly birthdays. The value for exclusive breastfeeding among 5-month olds might therefore be more representative for exclusive breastfeeding rate for the first 6 months as the infants received complementary diet between the ages of 5 and 6 months. This has also been seen in other European countries with high initiation rates [23]. There are numerous benefits of long breastfeeding duration and it has even been associated with lower adiposity at 15 years of age [24]. Therefore, more attention has to be given to increase breastfeeding rates in accordance to WHO recommendations [12]. In the present study partial breastfeeding duration was negatively associated with both Hb and MCV in boys, and exclusive breastfeeding for 5 months or longer was also associated with lower Hb level, although within the normal range, this might support the suggestion about reconsideration of the optimal length of exclusive breastfeeding [25]. In the previous Icelandic study, longer duration of breastfeeding was positively associated with iron status. This can be explained by the change in the total diet. The main substitute for breast-milk in the previous study was regular cow's milk, but in the present study it was iron-fortified formula. Furthermore, low iron content of breast-milk and an insufficient additional source of iron in the diet have been shown to affect iron status negatively [26, 27]. Iron concentration of breast-milk has though been suggested to increase during the weaning period [28]. The breast-milk as such does not seem to be the important component in this context but the total diet; sufficient iron intake from the complementary diet and the substitute for breastfeeding, that is, milk or formula, are the most important factors for good iron status in infancy. 

Meat consumption was most strongly independently associated with Hb in the present study's multiple regression analysis. This association is consistent with the 1995–1997 study, and meat consumption did not differ between the two studies. Meat is an important source of iron and a known enhancer of good iron status [4–6], and it apparently affects iron status even though meat consumption is very low in this age group. 

Another factor negatively associated with iron status, which was consistent in both studies, was growth rate. In the present study, iron-depleted children had markedly faster weight and length gain than non-iron-depleted ones. This difference was also seen in the 1995–1997 study between iron-deficient children and non-iron-deficient ones [7]. Furthermore, a study from the USA showed that 20% of overweight toddlers had ID versus 7% of normal-weight toddlers [29]. Faster growth during the first year has been proposed to affect iron status negatively [9] and BMI positively [30] at 6 years of age. This is probably attributed to the higher iron demands from a higher growth velocity [31]. Iron requirements are relatively the greatest during the rapid growth of infancy, especially from 6 months to 2 years of age, since full-term infants of non-iron-deficient mothers are born with sufficient iron storage to last for the first 4–6 months of life [18]. However, chronic iron deficiency over a long period has been shown to negatively affect growth in developing countries and some vulnerable groups in developed countries [10, 32, 33]. Limitations to the present study might be the incomplete data set for 31 of the 141 participants and lack of information on iron status at birth. However, iron status or other measures such as anthropometry and social status did not differ between those with complete and incomplete dietary data sets. In healthy full-term infants of normal birth weight it is unlikely that iron status at birth is influencing the status at the age of 12 months. Thus these possible limits are unlikely to influence the results and conclusion of the present study.

Iron stores were worse among boys; their SF levels were significantly lower than in girls and 9.9% of boys were below the cut-off values versus 1.5% of girls, which can not be attributed simply to differences in growth rate. Dietary factors and growth had more impact on iron status in boys than in girls (Table 4). A gender difference in SF level has also been seen in other studies, and Domellöf et al. concluded that it was possibly attributed to difference in iron metabolism because of hormonal differences between the sexes [34]. Furthermore, a higher proportion of boys were breastfed, and they had lower intake of iron-fortified formula at 12 months of age compared with girls in the present study, which might also partly explain the gender difference in iron status.

## 5. Conclusion

Iron status among 12-month-old Icelanders has improved enormously since the previous infant study. The largest alteration in the infants' diet between the two studies is the replacement of regular cow's milk by iron-fortified formula. The study demonstrates altered associations between iron status indices and food when the diet is changed. The lower intake of cow's milk deleted former association with ferritin. The association with duration of breastfeeding, although weak changed from being positive in the former cohort study to being negative in the present study. The effect of breastfeeding may mainly be derived from the other food used, that is, milk or formula, but not the breastfeeding as such. The findings also show the effect and importance of seeking solutions for public health problems with better dietary advice.

##  Conflict of Interests

The authors declare no conflict of interests.

## Figures and Tables

**Table 1:** Infant and parental characteristics of the subjects.

*Variable*	Boys	Girls
	Mean (SD)	Mean (SD)
No. of subjects [*n* (%)]	73 (51.8)	68 (48.2)
Infant characteristics		
Firstborn [*n* (%)]	20 (30.3)	24 (35.8)
Birth weight (g)	3807 (360)	3750 (369)
Birth length (cm)	52.2 (1.52)	51.7 (1.77)
Birth head circumference (cm)	36.2 (1.22)	35.4 (1.14)*
Weight at 6 mo (kg)	8.4 (0.7)	7.9 (0.75)*
Length at 6 mo (cm)	70.1 (1.7)	67.9 (1.7)*
Weight at 12 mo (kg)	10.37 (1.0)	9.76 (0.99)*
Length at 12 mo (cm)	78.0 (2.25)	76.17 (2.60)*
Hb at 12 mo (g/L)	120.96 (8.19)	120.28 (8.28)
MCV at 12 mo (fl)	76.71 (3.41)	77.69 (3.09)
log SF at 12 mo (*μ*g/L)	3.31 (0.63)	3.57 (0.46)*
Parental and socioeconomic characteristics		
Mother's BMI (kg/m^2^)	24.3 (6.09)	24.6 (5.38)
Father's BMI (kg/m^2^)	26.4 (4.14)	25.1 (5.60)
Mother's age (years)	32.0 (5.25)	30.3 (4.58)
Father's age (years)	34.1 (6.13)	33.4 (5.22)
Mother's ≥12 y of schooling [*n* (%)]	56 (82.4)	57 (86.4)
Father's ≥12 y of schooling [*n* (%)]	51 (77.3)	51 (78.5)
Smoker in the household [*n* (%)]	14 (21.5)	13 (20)
Reside in capital and surroundings [*n* (%)]	60 (62.5)	60 (60)
Marital status (married/partnership) [*n* (%)]	63 (96.9)	62 (98.4)

*Significantly different from boys. *P* ≤ 0.05 (indepentent *t*-test).

^†^Median and interquartile range (IQR).

**Table 2:** Intake of selected foods among boys and girls at 6, 9, and 12 months of age (*n* = 141 at 6 months, *n* = 122 at 9 months, and *n* = 110 at 12 months).

	Boys		Girls	
	%	Mean (SD) g/day	%	Mean (SD) g/day
*6 months*				
Partial breastfeeding	77.9		71.6	
Exclusive breastfeeding	11.1		3	
Iron fortified formula	21.9		19.8	
Whole milk	3.3		1.7	
*9 months*				
Iron-fortified formula	59.7	44.2 (243.7)*	68.3	48.8 (173.0)*
Whole milk	33.9	0 (14.7)*	36.7	0 (27.5)*
Breast milk^†^	50	21.7 (287.6)*	50	6.5 (297.5)*
Fruit & vegetables^‡^	100	99.0 (61.5)	98.3	89.5 (48.2)
Porridge	77.4	31.6 (33.1)	85	30.2 (26.0)
Dairy products^§^	50	2.9 (56.5)*	55	3.8 (58.3)*
Meat	79	8.2 (21)*	70	10.0 (26.9)*
*12 months*				
Iron-fortified formula	53.4	56.2 (222.6)*	75	212.9 (193.3)^||^
Whole milk	56.9	11.8 (67.7)*	51.9	6.7 (51.3)*
Breast milk^†^	26.5	0 (25.3)*	13.4	0 (0)^1, 5^
Fruits & vegetables^‡^	100	83.7 (51.3)	94.2	71.65 (46.2)
Porridge	82.8	78.6 (79.9)	92.3	93.0 (83.7)
Dairy products^§^	72.4	78.5 (81.1)	90.4	90.3 (69.7)
Meat	100	39.4 (39.5)	94.2	30.0 (26.3)

*Median and interquartile range (IQR).

^†^Mean values include nonbreastfed infants.

^‡^The food group includes infant purees and fresh fruits and vegetables. Percentage represents infants receiving either fruits or vegetables.

^§^Dairy products are milk products and cheese, excluding drinking milk.

^||^Significantly different from boys (Mann Whitney *U* test; *P* ≤ 0.05).

**Table 3:** Intake of selected nutrients among boys and girls at 9 and 12 months of age as an average intake over a 3-day period (*n* = 122 at 9 months and *n* = 110 at 12 months).

	*Boys*	*Girls*	
	Mean (SD)	Mean (SD)	
*9 months*			RDI (6–11 mo)
Energy (kJ/kg)	344.7 (82.4)	349.4 (83.6)	355
Protein (g/kg)	2.63 (0.89)	2.46 (0.87)	1.1
Vitamin C (mg/d)	67.2 (32.3)	71.6 (28.3)	20
Vitamin D (*μ*g/d)	9.7 (6.2)	9.4 (9.9)*	10
Vitamin A (RJ/d)	854.9 (678.1)*	947.6 (584.6)	300
Zinc (mg/d)	3.32 (1.78)	3.23 (1.42)	5
Iron (mg/d)	6.28 (3.19)	6.27 (2.73)	8
Calcium (mg/d)	510.8 (242.2)	488.2 (212.7)	540
*12 months*			RDI (12–23 mo)
Energy (kJ/kg)	351.1 (74.7)	353.8 (80.4)	355
Protein (g/kg)	3.04 (0.99)*	3.03 (0.95)	1.0
Vitamin C (mg/d)	58.1 (25.0)	60.2 (32.1)	25
Vitamin D (*μ*g/d)	9.1 (6.8)	8.3 (5.2)	10
Vitamin A (RJ/d)	785.3 (999.0)*	768.8 (492.1)^†^	300
Zinc (mg/d)	3.91 (2.01)	2.86 (2.28)*	5
Iron (mg/d)	6.82 (3.97)	5.77 (1.97)*	8
Calcium (mg/d)	565.2 (238.6)	576.0 (186.3)	600

* Median and interquartile range (IQR).

^†^ Significantly different from boys (Mann Whitney *U* test; *P* ≤ 0.05).

**Table 4:** Multiple regression analysis of weight growth and food factors influencing change in iron status indices.

Dependent variable At 12 months	Independent variable		Boys			Girls	
			95% Conf interval of B			95% Conf interval of B	
		B	Lower Bound	Upper Bound	B	Lower Bound	Upper Bound
Log SF	Length gain, cm (0–12 months)	−0.0823	−0.150	−0.014	—	—	—
	Weight gain, 100 g (0–6 months)	−0.0201	−0.040	−0.001	−0.0122	−0.027	0.003
	Weight gain, 100 g (0–12 months)	−0.0174	−0.032	−0.003	0.00454	−0.016	0.007
	Formula g/per day at 12 months	0.00077	−0.000062	0.0016	0.00077	0.00019	0.0014
	Porridge g/per day at 9 months	—	—	—	0.0124	0.002	0.023
	Bread g/per day at 9 months	—	—	—	−0.0113	−0.017	−0.005

Hb	Meat intake g/per day at 9 months	0.127	0.043	0.211	—	—	—
	Breastfeeding duration (mo)	−0.919	−1.473	−0.365	—	—	—
	Formula g/per day at 12 months	0.0145	0.004	0.025	—	—	—
	Fruit intake g/per day at 12 months	—	—	—	0.0384	0.009	0.068
	Dairy intake g/per day at 12 months	—	—	—	0.0319	0.003	0.061
	Iron intake mg/per day at 9 months	0.879	0.224	1.534	—	—	—

MCV	Breastfeeding duration (mo)	−0.261	−0.515	0.003	—	—	—
	Iron intake mg/per day at 12 months	—	—	—	0.297	0.061	0.534
	Vitamin C intake mg/per day at 9 months	—	—	—	0.0359	0.008	0.064

Adjusted for birth weight.

—No association found.
